# Neutral lipid storage disease with myopathy and myotonia associated to pathogenic variants on PNPLA2 and CLCN1 genes: case report

**DOI:** 10.1186/s12883-023-03195-6

**Published:** 2023-04-27

**Authors:** João Igor Dantas Landim, Ian Silva Ribeiro, Eduardo Braga Oliveira, Hermany Capistrano Freitas, Lara Albuquerque Brito, Isaac Holanda Mendes Maia, Daniel Gurgel Fernandes Távora, Cleonisio Leite Rodrigues

**Affiliations:** 1 Clinical Neurology Department From Hospital Geral de Fortaleza, Ceará, Brazil; 2 Neuromuscular Unit of Neurology Department From Hospital Geral de Fortaleza, Ceará, Brazil; 3 Clinical Neurophysiology of Neurology Department From Hospital Geral de Fortaleza, Ceará, Brazil; 4 Radiology Unit From Hospital Geral de Fortaleza, Ceará, Brazil

**Keywords:** Neutral lipid storage disease with myopathy, Thomsen’s congenital myotonia, Myopathy, Myotonia, Jordan’s anomaly, PNPLA2, CLCN1

## Abstract

**Background:**

Neutral lipid storage disease with myopathy (NLSD-M) is an autosomal recessive disease that manifests itself around the 3rd to 4th decade with chronic myopathy predominantly proximal in the shoulder girdle. Clinical myotonia is uncommon. We will report a rare case of association of pathogenic variants on PNPLA2 and CLCN1 genes with a mixed phenotype of NLSD-M and a subclinical form of Thomsen’s congenital myotonia.

**Case presentation:**

We describe a patient with chronic proximal myopathy, subtle clinical myotonia and electrical myotonia on electromyography (EMG). Serum laboratory analysis disclosure hyperCKemia (CK 1280 mg/dL). A blood smear analysis showed Jordan’s anomaly, a hallmark of NLSD-M. A genetic panel was collected using next-generation sequencing (NGS) technique, which identified two pathogenic variants on genes supporting two different diagnosis: NLSD-M and Thomsen congenital myotonia, whose association has not been previously described.

**Conclusions:**

Although uncommon, it is important to remember the possibility of association of pathogenic variants to explain a specific neuromuscular disease phenotype. The use of a range of complementary methods, including myopathy genetic panels, may be essential to diagnostic definition in such cases.

## Background

Neutral lipid storage disease with myopathy (NLSD-M) is a rare autosomal recessive disease [1, 2] caused by mutations in the *Patatin Like Phospholipase Domain Containing 2* (*PNPLA2*) gene which encodes adipose triglyceride lipase (ATGL). This enzyme participates of the intracellular triglyceride hydrolysis in the cellular cytoplasm thus a complete or partial loss of the enzymatic activity affects the energetic metabolism leading to intracellular accumulation of lipid vacuoles containing triacylglycerol (TAG) and cell damage [1, 3, 4].

Clinical findings of NLSD-M are related to impairment of striated skeletal and cardiac musculature [5]. The typical age of symptoms onset is in the second or third decade, starting with slow progressive proximal limb weakness and atrophy, especially in shoulder girdle (70% of the cases) [6], although less pronounced pelvic girdle and distal musculature involvement are commonly present [6]. Cardiac pathology can occur lately in half of patients, usually observed in the fourth decade of life [6]. Hepatomegaly, diabetes mellitus and hypertriglyceridemia can also occur [7, 8]. Asymptomatic hyperCKemia may be an initial manifestation with development of myopathy years later [9]. In contrast, orofacial and ocular musculature dysfunction, dysphagia, and respiratory failure are usually absent.

Pathogenic variants on CLCN1 gene are associated to congenital myotonia and the pattern of genetic inheritance can be autosomal recessive (eg. Becker disease) or autosomal dominant (eg. Thomsen disease). The hallmark of these conditions is the myotonic phenomenon, a delayed relaxation of skeletal muscles after voluntary contraction, that can be associated with painful muscle cramps, muscle hypertrophy and proximal weakness. Becker disease presents with late-onset symptoms, moderate to severe myotonia and transient proximal muscle weakness that can become permanent. On the other hand, Thomsen disease has an earlier onset, with mild symptoms and permanent muscle weakness is usually absent. Furthermore, pathogenic variants related to Thomsen disease have reduced penetrance and variable phenotype presentation [10].

Here, we describe a patient with chronic proximal myopathy, subtle clinical myotonia and electrical myotonia on electromyography (EMG) related to both pathogenic variants on PNPLA2 and CLCN1 genes. This association of NLSD-M and a subclinical form of Thomsen’s congenital myotonia not previously described was made through clinical, EMG and genetic findings coexisting in the same patient.

## Case presentation

A 42 years-old female patient was admitted to a reference center for Neuromuscular Disorders in northeast of Brazil with a 7 year history of progressive proximal upper limbs weakness. Five years after symptoms onset, she also noticed proximal lower limbs and distal upper limbs weakness associated to proximal muscle atrophy, specially of shoulder girdle. Strenuous exercise and cold exposure induces myalgia and worsening of weakness.

There were no complaints of fluctuating symptoms, diplopia, eyelid ptosis, dysphagia, dysphonia, dysarthria, dyspnea or abnormal urine color. Past medical history was negative for other conditions as well as substance abuse. Family health history was also negative for neuromuscular diseases.

**Fig. 1:**
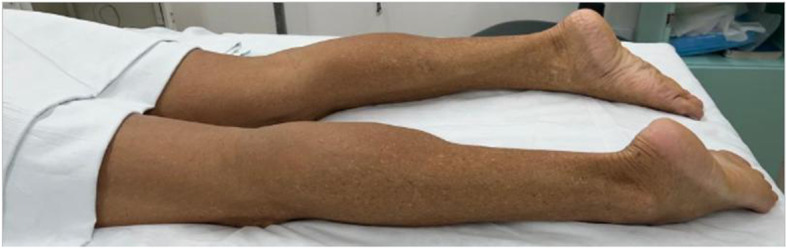
The calf´s muscle had a mild-moderate level of pseudohypertrophy

Clinical evaluation confirmed proximal weakness of four limbs, moderate on pelvic girdle and severe on shoulder girdle, the calf’s muscle had a mild level of pseudohypertrophy with increased tonus (Fig. 1). with atrophy and handgrip myotonia. Fasciculations, joint contractures or sensory impairment were not present. Deep tendon reflexes were globally reduced.

Serum laboratory analysis disclosure hyperCKemia (CK 1280 mg/dL) and excluded others abnormalities (electrolytes disturbance, thyroid, kidney and liver dysfunction), infectious diseases (retrovirus, syphilis, hepatitis B and C) and serum biomarkers of collagenosis or inflammatory myopathies. Serum protein electrophoresis was normal.

**Fig. 2:**
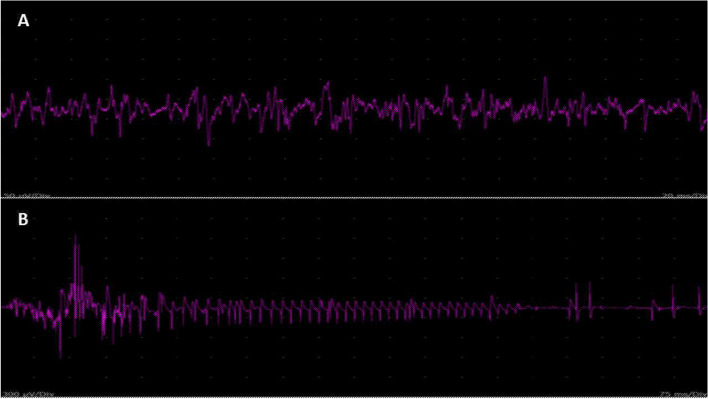
**a**. Myopathic motor recruitment (early recruitment, small-amplitude and short-duration of MUAPs, short-polyphasics MUAPs) recorded in the left deltoid muscle. **b**. Myotonic discharge (waxing and waning of both amplitude and frequency) recorded in the thoracic paravertebral muscle

**Fig. 3:**
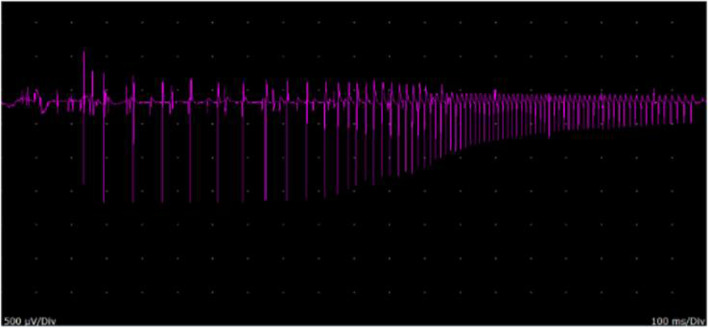
Myotonic discharge (waxing and waning of both amplitude and frequency) recorded in the left extensor digitorum communis muscle

Nerve conduction studies (NCS) and needle EMG were performed, which showed normal motor and sensory nerve conductions studies parameters and myopathic pattern: increased and early motor recruitment, decreased duration and amplitude of motor unit potentials (MUP) with short polyphasic potentials. The EMG abnormalities were more evident on proximal upper limb muscles and with a mosaic pattern (myopathic/normal) on lower limb muscles. Furthermore, signs of membrane instability (fibrillations and positive sharp waves) were identified on upper and lower limbs and paravertebral musculature as well as electrical myotonia in some muscles of upper and lower limbs and paravertebral musculature (Figs. 2 and 3). Slow repetitive nerve conduction study was normal.

**Fig. 4:**
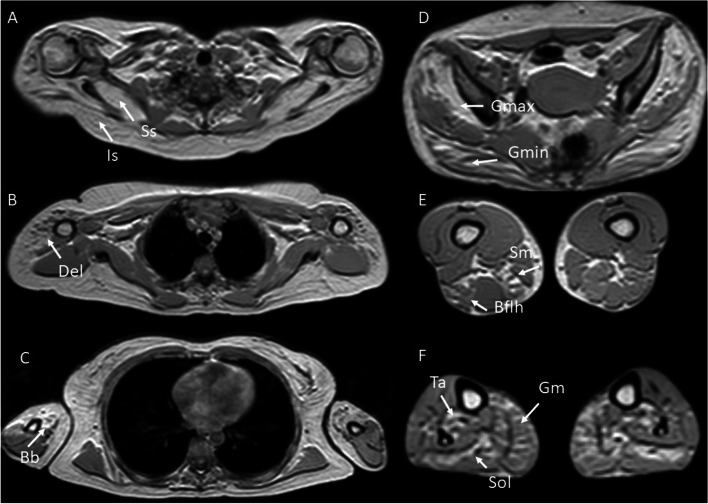
T1 weighted images on axial plane. Symmetrical muscle Fat replacement is shown in the supraspinatus and infraspinatus (Ss and Is in **A**), deltoid (Del in **B**), biceps brachii (Bb in **C**), gluteus minimus and gluteus maximus (Gmin and Gmax in **D**), semimembranosus and biceps femoris long head (Sm and Bflh in **E**), soleus, medial head of gastrocnemius and tibialis posterior (Sol, Gm and Ta in **F**)

Patient underwent a whole-body 1,5 Tesla magnetic resonance image to assess the pattern of muscle impairment. Fat replacement degree was measured using modified Mercuri scale by Fischer et al., 2008 [11] – Fig. 4. Then, we found the following pattern of fat replacement: soleus, medial head of gastrocnemius and posterior tibialis (grade 2), semimembranosus and long head of biceps femoris (grade 2), gluteus minimus (grade 4), gluteus maximus (grade 3), deltoid (grade 2), biceps brachii and brachii (grade 4), supraspinatus and infraspinatus (grade 4). It also showed a hyperintense signal on STIR sequence involving soleus, medial head of the gastrocnemius and posterior tibial, showing signs of muscle edema.

**Fig. 5:**
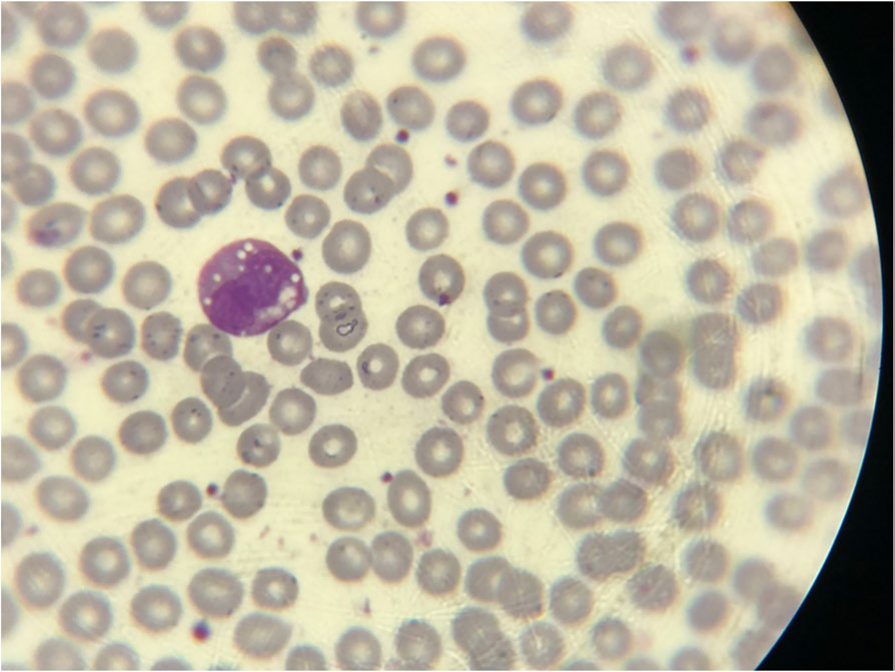
Leucocyte presenting lipid droplets (Jordans’ anomaly); optic microscopy, Wright-Giemsa-leishman, 100 ×

Given the initial suspicion of inherited metabolic myopathy, a genetic myopathy panel was collected using the NGS technique, which identified pathogenic variants on Gene *NM_020376.4(PNPLA2):c.792del(p.Leu264fs)* and *NM_000083.3(CLCN1):c.1453A* > *G(p.Met485Val)* genes supporting two different diagnosis: NLSD-M and Thomsen congenital myotonia. Peripheral blood smear analysis showed as Jordan’s anomaly (Fig. 5), a hallmark of NLSD-M.

We also performed a multisystemic evaluation with electrocardiogram, transthoracic echocardiogram, abdominal ultrasound, ophthalmological evaluation and lipid and glycemic profile that were normal.

## Discussion and conclusions

This case report presents a unique case of an association NLSD-M and a subclinical form of Thomsen’s congenital myotonia, with no other similar case reported in literature for our knowledge. Initially, the suspicion of a genetic etiology for this condition arose due to the chronic and progressive course of proximal myopathy without an obvious secondary acquired cause. Is important to notice that a negative familial history does not put away a genetically based etiology, especially due the possibility of a de novo mutation, incomplete inheritance and lack of information about the actual degree of kinship between family members.

In the presence of paravertebral electrical myotonia and exercise intolerance is essential to exclude metabolic myopathies, mainly Pompe disease and myotonic dystrophy type 2 as possible etiologies. However, this case report shows other important differential diagnosis of metabolic myopathy: NLSD-M, in which an asymmetric weakness more evident on shoulder girdle raises suspicion. A hallmark of this condition that is present on 100% of cases is the presence of triacylglycerol (TAG) vacuoles in leukocytes cytoplasm evident on peripheral blood smear, denominated Jordan’s anomaly [1, 12]. Thus, this simple laboratory test can be used as a screening evaluation for young patients with isolated hyperCKemia, aiming for an early diagnosis of NLSD-M [1, 12].

In this case, we found two rare pathogenic variants in the same patient that explained all the clinical and EMG findings. Thus, although uncommon, it is important to remember the possibility of association of pathogenic variants to justify a specific neuromuscular disease phenotype. A broad genetic panel evaluation was essential for diagnostic definition.

It is important to report the relevance of genetic sequencing, especially in neuromuscular diseases where genetic etiologies are very common. Since the beginning of the Human Genome Project and the improvement of NGS in 2005 there has been a revolution in the field of neurogenetics and changes in the diagnostic approach in neuromuscular diseases. Clinical, EMG and laboratory aspects should be taken into consideration for a better phenotype-genotype association, as in our case report in which we found the double phenotype as the main hypothesis to explain clinical manifestations. In this scenario, greater access to genetic testing can be extremely important in clinical practice for diagnostic definition, therapeutic decision, prognostic evaluation, genetic counseling and even for recruitment in clinical trials [13].

## Data Availability

The datasets used and/or analyzed during the current study are available from the corresponding author on reasonable request.

## Abbreviations

NLSD-M: Neutral lipid storage disease with myopathy
PNPLA2: Patatin like phospholipase domain containing 2
ATGL: Adipose triglyceride lipase
TAG: Triacylglycerol
EMG: Electromyography
NCS: Nerve conduction studies
MUP: Motor unit potentials
NGS: Next-generation sequencing
